# Gas‐Assisted Spray Coating of Perovskite Solar Cells Incorporating Sprayed Self‐Assembled Monolayers

**DOI:** 10.1002/advs.202104848

**Published:** 2022-02-09

**Authors:** Elena J. Cassella, Emma L. K. Spooner, Timothy Thornber, Mary E. O'Kane, Thomas E. Catley, James E. Bishop, Joel A. Smith, Onkar S. Game, David G. Lidzey

**Affiliations:** ^1^ Department of Physics and Astronomy University of Sheffield Hicks Building, Hounsfield Road Sheffield S3 7RH UK; ^2^ Department of Physics University of Oxford Clarendon Laboratory Parks Road Oxford OX1 3PU UK

**Keywords:** air‐knife, gas‐quenching, perovskite solar cells, scalable fabrication, self‐assembled monolayers, spray coating

## Abstract

Self‐assembled monolayers (SAMs) are becoming widely utilized as hole‐selective layers in high‐performance p‐i‐n architecture perovskite solar cells. Ultrasonic spray coating and airbrush coating are demonstrated here as effective methods to deposit MeO‐2PACz; a carbazole‐based SAM. Potential dewetting of hybrid perovskite precursor solutions from this layer is overcome using optimized solvent rinsing protocols. The use of air‐knife gas‐quenching is then explored to rapidly remove the volatile solvent from an MAPbI_3_ precursor film spray‐coated onto an MeO‐2PACz SAM, allowing fabrication of p‐i‐n devices with power conversion efficiencies in excess of 20%, with all other layers thermally evaporated. This combination of deposition techniques is consistent with a rapid, roll‐to‐roll manufacturing process for the fabrication of large‐area solar cells.

## Graphical abstract

Carbazole‐based self‐assembled monolayers are becoming a dominant hole‐transporting layer in p‐i‐n perovskite solar cells, combining stability, efficiency, and low‐cost. Here, spray coating and airbrush pen coating of MeO‐2PACz are used to fabricate high‐quality transport layers. This is combined with gas‐quenched spray‐coated perovskite layers, to realize solar cells with power conversion efficiencies in excess of 20%.

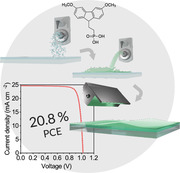

## 1 Introduction

Organic–inorganic metal halide perovskite photoabsorbers have enabled the development of single‐junction perovskite solar cells (PSCs) with power conversion efficiencies (PCEs) of up to 25.5%.^[^ ^1^ ^]^ Further gains in device efficiency (toward the theoretical thermodynamic efficiency limit) are anticipated via the further suppression of nonradiative recombination pathways within PSCs, both within the bulk perovskite material and at the transport layer interfaces.^[^ ^2^, ^3^, ^4^ ^]^ Losses at such interfaces have generally limited the efficiency of p‐i‐n (“inverted”) architectures compared to n‐i‐p PSCs. However, p‐i‐n devices offer promising operational stability, competitive large‐area performances, and compatibility with silicon bottom cells in perovskite/Si tandem devices.^[^ ^5^, ^6^, ^7^, ^8^ ^]^ For this reason, the development of surface passivation and interface management strategies have been the focus of recent efforts to enhance p‐i‐n PSC device performance.^[^ ^9^, ^10^ ^]^ One material system of particular interest are self‐assembled monolayers (SAMs) that comprise carbazole moieties and phosphonic acid tail groups, with such materials capable of creating practically lossless hole‐transport interfaces.^[^ ^11^, ^12^ ^]^ SAMs, therefore, represent a simple and low‐cost PSC technology, with state‐of‐the‐art inverted devices demonstrating PCEs approaching 23%^[^ ^13^ ^]^ and perovskite/Si tandems surpassing 29%.^[^ ^14^ ^]^

To drive PSC technology toward commercialization, it is increasingly important to develop new perovskite and charge‐transporting materials that combine high‐performance, enhanced operational stability, low‐cost and the ability to be deposited using scalable techniques.^[^ ^15^ ^]^ In n‐i‐p PSCs, the widely used hole‐transport layer (HTL) spiro‐OMeTAD has been a bottleneck to the development of large‐area devices due to a combination of thermal and dopant instability and high materials cost.^[^ ^16^, ^17^ ^]^ Other commonly used p‐i‐n HTLs also have drawbacks; for example the conjugated polymers poly[bis(4‐phenyl)(2,4,6‐trimethylphenyl)amine] (PTAA) and poly(N,N′‐bis‐4‐butylphenyl‐N,N′‐bisphenyl)benzidine (poly‐TPD) require costly synthesis. NiO*_x_* has been used to create spray‐based minimodules,^[^ ^18^ ^]^ however, it typically requires high‐temperature sintering steps, which limits its end‐use applications. The polymer blend HTL poly(3,4‐ethylenedioxythiophene) polystyrene sulfonate (PEDOT:PSS) is known to be hygroscopic and can cause device instability.^[^ ^19^ ^]^ Recently, carbazole‐based SAMs have emerged as a new class of HTLs for PSCs. These materials present an exciting new opportunity to combine scalability and stability with significantly reduced materials cost compared to the current state‐of‐the‐art devices. In particular, devices based on the SAMs MeO‐2PACz (([2‐(3,6‐dimethoxy‐9H‐carbazol‐9‐yl)ethyl]phosphonic acid) and 2PACz ([2‐(9H‐carbazol‐9‐yl)ethyl]phosphonic acid) have been fabricated by both spin‐ and dip coating, with 2PACz also used in PSCs fabricated via slot‐die coating and used to create minimodules.^[^ ^20^ ^]^

The development of appropriate high‐throughput PSC fabrication processes will require deposition technologies that can be used to fabricate thin films at high volume and high speed, with this being a critical component of a practical commercialization process.^[^ ^21^ ^]^ Here, spray coating enables both unparalleled linear deposition speeds of up to 12 m min^–1^,^[^ ^22^ ^]^ and also permits deposition over nonplanar surfaces.^[^ ^23^, ^24^, ^25^, ^26^ ^]^ This has driven a growing interest in the utilization of spray coating to deposit all active layers in PSC devices. However, the performance of spray‐fabricated PSCs and modules has not yet matched those prepared by other scalable techniques.^[^ ^27^, ^28^ ^]^ For example, state‐of‐the‐art spray‐cast PSCs presently exhibit PCEs of only around 20% and have required postdeposition treatments which may limit their potential for roll‐to‐roll processing, such as submersion into an antisolvent bath^[^ ^29^ ^]^ or exposure to a vacuum to induce supersaturation.^[^ ^30^ ^]^ Furthermore, dewetting effects can occur during spray‐deposition, which generate morphological defects and limit the performance of large‐area spray‐coated PSCs.^[^ ^31^ ^]^ Such effects can be pronounced when using hydrophobic transport layers typical in p‐i‐n devices. Here, we note that perovskite crystallization dynamics can be controlled using gas‐assisted techniques in which a high‐pressure gas‐jet from an air‐knife is directed across a drying film. This technique (first explored in 2014 by Huang et al.^[^ ^32^ ^]^) was first used to dry spin‐coated MAPbI_3_ films (achieving peak PCEs of 17%) and then applied to other perovskite compositions.^[^ ^33^ ^]^ Following this, gas‐assisted crystallization has quickly gained interest and has been utilized in films that are drop‐cast,^[^ ^34^, ^35^ ^]^ bar,^[^ ^36^, ^37^, ^38^ ^]^ blade,^[^ ^39^, ^40^, ^41^, ^42^, ^43^, ^44^, ^45^, ^46^, ^47^ ^]^ and inkjet‐printed,^[^ ^48^ ^]^ and has been used to create slot‐die PSCs and modules,^[^ ^20^, ^49^, ^50^, ^51^, ^52^, ^53^, ^54^, ^55^ ^]^ tandem‐devices^[^ ^56^ ^]^ and light‐emitting diodes (LEDs).^[^ ^57^ ^]^ Recently, gas‐assisted, spin‐coated, passivated FA_0.80_MA_0.15_Cs_0.05_PbI_2.55_Br_0.45_ devices have been fabricated with a record PCE of 23.6%.^[^ ^58^ ^]^ Therefore, it is clear that the use of gas‐quenching represents a potentially robust post‐treatment in a practical PSC manufacturing process.

In this paper, we combine three key technologies described above, namely high‐performing SAM hole‐transport layers, ultrasonic spray coating, and gas‐assisted quenching to fabricate high‐efficiency p‐i‐n PSCs. We firstly deposit an MeO‐2PACz SAM transport layer using an ultrasonic spray‐deposition route which is combined with a spin‐coated MAPbI_3_ layer, realizing devices with an efficiency of over 20%. We then develop a novel gas‐assisted spray processing (GASP) protocol to spray‐cast highly crystalline and uniform MAPbI_3_ perovskite films on top of a hydrophobic MeO‐2PACz SAM transport layer. Finally, we spray coat both the SAM HTL and the MAPbI_3_ perovskite, realizing devices with a stabilized efficiency of 20.5%. We believe this work further demonstrates the promise of spray coating as an industrially viable, scalable deposition technique for low‐cost PSC manufacture.

## 2 Results and Discussion

### 2.1 Methodology

**Figure 1:**
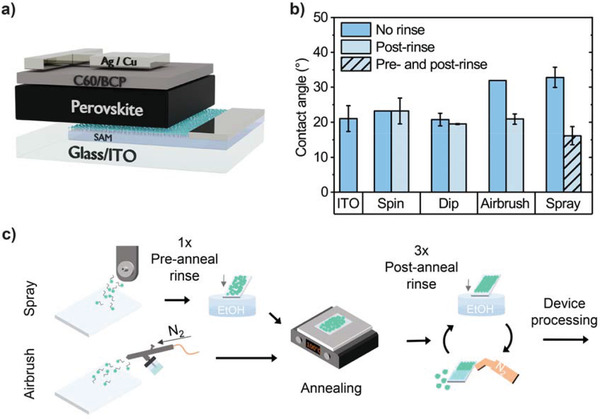
a) Illustration of the p‐i‐n device structure used. b) Initial contact angle for 2‐methoxy ethanol (2‐ME) on clean ITO and MeO‐2PACz films deposited on ITO via each technique without rinsing (darker‐blue) and for post‐rinsed films (lighter‐blue). For spray‐coated MeO‐2PACz, we found that an additional prerinse, together with a postrinse significantly improved the 2‐ME contact angle (hatched‐area). c) A schematic of the optimized rinsing procedures for spray‐ and airbrush‐coated MeO‐2PACz.

All devices were fabricated on a pixelated indium tin oxide (ITO) anode unless otherwise stated. As we describe below, we explored airbrush‐, spray‐, dip‐, and spin‐coated MeO‐2PACz SAM transport layers. An MAPbI_3_ perovskite precursor solution was then deposited on top of the SAM HTL using either spin coating or gas‐assisted spray coating, with all spray and spin deposition processes performed in a nitrogen‐filled glovebox. Finally, devices were completed by the thermal evaporation of a C_60_/bathocuproine (BCP)/silver cathode. **Figure** **1**a shows a schematic of the resultant p‐i‐n device stack. Full details of all materials, fabrication techniques and processes used are described in the Experimental Methods.

### 2.2 Developing Spray‐Coated Self‐Assembled Monolayers

We have investigated two scalable deposition techniques to deposit an SAM HTL layer, namely ultrasonic spray coating (referred to henceforth as “spray”) and spray coating with an airbrush pen (denoted here as “airbrush”). Spray coating is a relatively sophisticated technique that typically requires careful control of parameter space to facilitate the deposition of high‐quality layers.^[^ ^59^ ^]^ In contrast, airbrush pen spray coating is a more straightforward and widely accessible process that is ideally suited to deposit a SAM. Here, it is simply necessary to ensure that all parts of a surface become coated with the SAM material, with the phosphonic acid component of each molecule forming (upon annealing) a covalent bond with oxide vacancies at the substrate surface.^[^ ^60^ ^]^

We firstly discuss the optimization of the deposition of the MeO‐2PACz SAM HTL. Here, we compare the fabrication of films and the performance of devices in which the SAM was deposited using spray‐ and airbrush deposition, with those in which it was deposited by spin coating or dip coating from a 1 or 0.1 mmol ethanol solution respectively, with the substrate held at room temperature in all cases.^[^ ^12^ ^]^ The SAMs were then annealed at 100 °C for 10 min. Our initial experiments demonstrated that to produce a uniform wet film across a substrate, it was necessary to make three sequential spray passes using an ultrasonic spray‐coater, with minimal solvent evaporation observed between each pass. Initial experiments indicated that if fewer spray‐passes or reduced solvent flow rates were used, then the performance of the resultant devices was widely distributed, indicating that the SAM did not uniformly coat the surface (see Figure S1, Supporting Information).

It is known that the SAMs used are hydrophobic (having a high contact angle) and can cause the dewetting of subsequently deposited layers. Indeed, the formation of a SAM on an ITO surface is known to be self‐limiting; once all molecular surface binding sites are occupied, additional molecules do not attach to the ITO but instead remain as unbound material. Here, additional unbound molecules result in an MeO‐2PACz film having a larger contact angle due to exposed phosphonic acid groups.^[^ ^61^ ^]^ We can therefore use contact angle measurements as a probe to explore the degree to which a complete SAM layer is formed without problems resulting from excess unbound materials. In our device studies, we deposited the MAPbI_3_ perovskite precursor from a 2‐methoxy ethanol solution (2‐ME) which has previously been shown to create efficient PSCs when used with a 2PACz hole‐extracting contact.^[^ ^20^ ^]^

Figure 1b shows the contact angle of a drop of 2‐ME solvent on top of annealed SAM films prepared via spin coating, dip coating, airbrush coating and spray coating. The figure also records the contact angle of 2‐ME on a clean ITO surface for reference. Interestingly, we find that the initial contact angle of the spray and airbrush‐coated MeO‐2PACz films were much higher for than for dip‐ or spin‐coated layers, being 32.8° and 31.9° versus 20.7° and 23.2°, respectively. In all cases, the contact angle of the SAM films was similar or larger than that of the clean ITO/glass control (21.0°). We also find a similar trend for contact angle measurements performed using a dimethylformamide: dimethyl sulfoxide (DMSO) solvent blend and deionized water (see Figure S2a,b, Supporting Information). We interpret this result in terms of an excess of MeO‐2PACz on the ITO surface when they are deposited using spray and airbrush techniques.

We found that dewetting of the perovskite precursor arising from this excess of MeO‐2PACz severely impacted device performance (see Figure S3, Supporting Information, and corresponding discussions). Previous research has described the removal of excess SAM material via rinsing, washing, or sonicating in the deposition solvent.^[^ ^62^, ^63^, ^64^ ^]^ Such rinsing processes are typically applied after thermal annealing (referred to henceforth as a “postrinse”), as they result in the formation of stronger, covalent bonds that attach the monolayer material to the surface, and thereby prevent the monolayer being unintentionally stripped from the surface. In our experiments, we “dip‐rinsed” the annealed SAM films in ethanol and then dried them under a flow of nitrogen, with this process performed three times. However, an additional process was also found to be needed as rinsing spray‐coated SAMs postannealing was alone not sufficient to fully alleviate wetting issues (see Figure S3a, Supporting Information). This was because it was observed that without any rinsing, or with only a postrinse, spray‐coated perovskite precursor inks *would not* coalesce to form a continuous film. We therefore also explored rinsing films *before they are annealed* in order to remove a greater degree of material from the (still wet) film; an approach recently established to remove aggregates and multilayers of spray‐coated gemini perfluorinated phosphonic acid SAMs deposited on top of ITO.^[^ ^65^ ^]^

In our experiments, we therefore explored the use of various numbers of “prerinse” steps, with this study guided by device optimization (Figure S3b, Supporting Information) with the optimized rinse/prerinse protocol developed in Figure 1c. It was found that a single prerinse, in combination with a postrinse, was beneficial when applied to films that had been spray‐cast, but instead was detrimental when applied to devices made from films deposited using an airbrush or spin coating. When spray‐coating SAMs, we believe that unbound excess material is removed by the first prerinse. This both improves wetting behavior, but also likely improves the charge transport at the perovskite: HTL interface by removing any material accumulated there that would otherwise hinder charge transfer. This initial prerinse results in a PCE increase likely due to improved extraction of charge carriers. With subsequent preannealing rinses however, we speculate that we begin to remove loosely bound material at the ITO surface (i.e., having lower denticity^[^ ^66^ ^]^). This causes a decrease in PCE due to the removal of regions of the monolayer leading to contact between the perovskite and the ITO, resulting in a poorly rectifying junction. For airbrush‐coated MeO‐2PACz, a smaller volume of deposited material results in a lower quantity of excess material and thus the prerinsing step immediately begins rinsing away regions of the loosely bound monolayer, leading to a drop in PCE. We suspect that the relative amounts of excess unbound or loosely bound material will vary between sample types (being dependent on the deposition conditions) and explains the different effects of rinsing that we see.

The effect of the rinsing processes on contact angle can be seen in Figure 1b. Here, it is evident that the contact angle of 2‐ME on the spray‐ and airbrush‐coated MeO‐2PACz films is reduced by the rinsing procedures to 16.2° and 20.8° respectively, with this value being comparable to, or lower than similar rinsed MeO‐2PACz films prepared by spin or dip coating. We attribute the greater reduction in contact angle upon rinsing for spray‐ and airbrush‐deposited layers as resulting from a larger degree of excess material being present at the surface which is then removed. We have attempted to explore changes in film roughness using atomic force microscopy (AFM) imaging as a result of coating with MeO‐2PACz films and the effect of the rinsing process on such coated films (Figure S4, Supporting Information). We find however that such effects are small (statistically insignificant), and we conclude that the thin MeO‐2PACz layers conformally coat the ITO surface,^[^ ^67^ ^]^ with the AFM being unable to resolve significant changes in roughness due to the polycrystalline nature of the ITO surface on which it is deposited.

**Figure 2:**
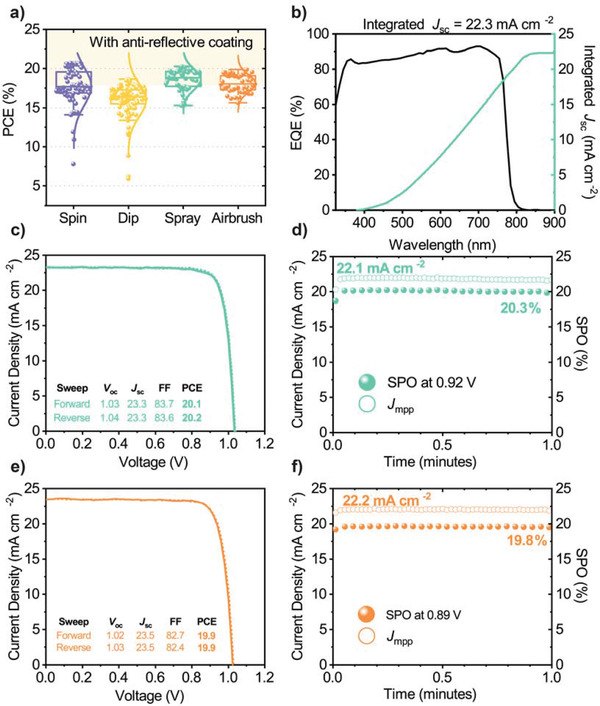
a) Device PCE for spin‐coated MAPbI_3_ devices fabricated on ITO substrates with a spin‐ (unrinsed, purple), dip‐ (postanneal rinsed only, yellow), spray‐ (pre‐ and post‐rinsed, green), and airbrush‐ (post‐rinsed only, orange) deposited MeO‐2PACz hole‐selective transport layer prepared as described in the text. b) External quantum efficiency, c) JV curve, and d) stabilized power output (SPO) for the best‐performing spray‐coated MeO‐2PACz device. e) JV curve and f) SPO for the best‐performing airbrush‐coated MeO‐2PACz device. Both champion devices incorporate an antireflective coating.

We have used the MeO‐2PACz SAM layers prepared using the optimized rinsing techniques described above (see Figure 1c and summarized in Table S1, Supporting Information) to fabricate PSC devices. Here, our reference (control) devices included an MAPbI_3_ active layer that was deposited using a gas‐assisted spin coating protocol as described in the Experimental Methods. The spread of device PCEs prepared via the different deposition routes is shown in **Figure** **2**a. Similar performances were realized for spin‐coated MAPbI_3_ devices incorporating an airbrush or spray‐coated MeO‐2PACz layer and the spin‐cast SAM controls. Here, the champion spray‐ and airbrush‐coated MeO‐2PACz devices achieved a PCE of 20.2% (20.3% stabilized) and 19.9% (19.8% stabilized) respectively (see current‐voltage (JV) curves and external quantum efficiency (EQE) data in Figure 2b–f). We note that a small mismatch observed between the *J*_sc‐EQE_ and *J*_sc‐JV_ (1 mA cm^–2^) falls within acceptable limits as discussed previously.^[^ ^68^, ^69^ ^]^ All average and champion performance metrics are tabulated in Tables S2a,b in the Supporting Information.

### 2.3 Developing an Air‐Knife Gas‐Quenching Process for Perovskites Deposited on Spin‐Coated SAMs

This section discusses the deposition and optimization of MAPbI_3_ perovskite films and PSCs using a gas‐assisted spray‐process (GASP). Here, such films are deposited on spin‐coated MeO‐2PACz SAMs on ITO substrates without any antireflective coating applied.

It is well understood that the performance of a PSC is a function of the quality of the crystalline perovskite layer. This is highly dependent on the nucleation and growth of crystal grains, with such nucleation triggered by the supersaturation of the precursor. Typically, this involves the use of an antisolvent during spin coating.^[^ ^70^ ^]^ However, to trigger nucleation during or after spray coating, researchers have also used an antisolvent bath method,^[^ ^29^, ^71^ ^]^ although the scalability of this processes is limited by the use of significant quantities of solvent that becomes gradually “contaminated” during any device run. Alternative approaches to induce supersaturation include thermal annealing,^[^ ^72^ ^]^ vacuum‐assisted solution processing,^[^ ^73^ ^]^ hot‐air blowing,^[^ ^74^ ^]^ and plasma‐treatments.^[^ ^22^ ^]^ Here, we investigate a GASP protocol in which an ambient temperature nitrogen gas‐jet from an air‐knife is blown at a surface that has been spray‐cast with a perovskite precursor solution, with the evaporation of the volatile casting‐solvent inducing supersaturation. For further information on gas‐quenching techniques we direct the reader to a recent review.^[^ ^75^ ^]^

In the GASP process developed, an ultrasonic spray‐head was moved linearly across the substrate surface, uniformly depositing an MAPbI_3_ precursor solution from a 2‐ME solvent. This highly volatile precursor system has previously been used to deposit MAPbI_3_ thin films,^[^ ^76^ ^]^ and has been used with 2PACz SAM layers to realize MAPbI_3_ devices with a PCE of 20.8%.^[^ ^20^ ^]^ In our process a short delay was included to allow the precursor droplets to coalesce (30 s), after which an air‐knife (blowing ambient temperature nitrogen at a pressure of 20 psi) was moved across the substrate. During this process, the distance between the surface and the air‐knife was maintained at around 2 cm. It was found that the application of the gas‐jet induced a rapid color change of the precursor film from yellow to dark brown. The entire process was performed inside a nitrogen‐filled glovebox at room temperature, with the films produced being annealed at 100 °C for 10 min. We illustrate the GASP procedure in Figure S5a–c in the Supporting Information.

**Figure 3:**
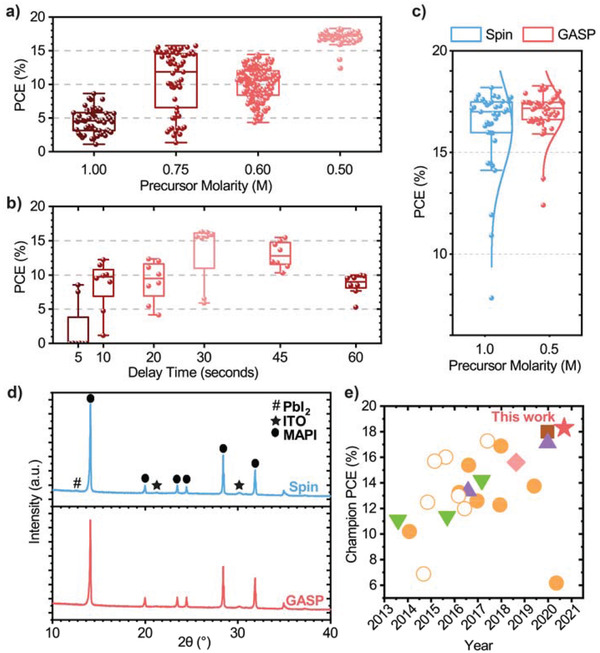
All devices are fabricated on ITO and do not include an antireflective coating. a) Photovoltaic performance of gas‐assisted spray‐processed (GASP) MAPbI_3_ devices as a function of the molarity of the 2‐methoxy ethanol precursor ink. b) Photovoltaic performance of GASP devices fabricated from a 0.5 m precursor ink with increasing delay time between deposition of the ink and application of a N_2_ gas‐jet. c) Photovoltaic device efficiency of MAPbI_3_ devices prepared using GASP under optimized conditions (0.5 m, 30 s delay, red) versus spin‐coated control devices (blue). d) X‐ray diffraction patterns for spin‐coated (blue) and GASP MAPbI_3_ (red) films. e) The development of spray‐cast p‐i‐n and n‐i‐p MAPbI_3_ devices over time; p‐i‐n (filled orange circles) and n‐i‐p (outlined orange circles) MAPbI_3_, p‐i‐n MAPbI_3−_ *_x_* Cl*_x_* (inverted green triangles), MAPbI_3−_ *_x_* Br*_x_*, Cs*_x_* FA_1−_ *_x_* PbI_3_, (brown square), and mixed cation (pink diamond). This work (pink star) demonstrates the highest power conversion efficiency for spray‐coated MAPbI_3_ perovskite solar cells achieved to date (more details can be found in Table S4, Supporting Information).

As part of our optimization process, we have explored the effect of the molarity of the spray‐cast precursor solution on device performance. We find that when the precursor was spray‐cast at a molarity of 1 m, the top surface of the film appeared highly crystalline however the quality of the bottom interface was extremely poor. We reach this conclusion from the cloudy appearance of such films (see Figure S3a, Supporting Information) which suggest poor contact with the underlying substrate caused by the presence of voids in the film. We believe that when the gas‐jet is applied to films spray‐cast from high concentration precursors, the evaporation of solvent proceeds more quickly than the solvent molecules can diffuse through the “wet” film. As a result, a uniform composition does not establish throughout the depth of the wet film, with regions closer to the surface becoming supersaturated and therefore undergoing rapid crystallization.^[^ ^77^ ^]^ This most likely results in dendritic crystal growth from the film surface, (a top‐down crystallization mechanism^[^ ^78^ ^]^) that is exacerbated by the application of the gas‐jet. This process has been previously explored in detail in films cast from DMSO‐containing precursors—with a solvent‐trapping phenomenon identified that leads to the formation voids in the film as the trapped solvent escapes upon annealing.^[^ ^53^, ^79^ ^]^ The results of this effect are illustrated practically in **Figure** **3**a, where it can be seen that device PCE increases as the precursor molarity is reduced.

As part of our optimization studies we have explored the effect of the “delay time” between spray‐coating the perovskite precursor solution and the application of the N_2_ gas‐jet. This is shown in Figure 3b where is can be seen that device efficiency is apparently optimized when deposited from a solution having a molarity of 0.5 m, where a delay of around 30 s is included between spray‐casting and the application of the gas‐jet. We discuss this effect in more detail below. Figure 3c summarizes the efficiency of MAPbI_3_ devices prepared using GASP under optimized conditions (0.5 m, 30 s delay). This is compared with control devices created using a gas‐assisted spin coating protocol in which a jet of N_2_ at around 20 psi was directed at the spinning substrate 6 s after the deposition of the perovskite precursor solution. Here, we find the champion efficiency of GASP and control devices to be 18.3% (17.0 ± 1.0%) and 18.2% (16.2 ± 2.1%) respectively. The close similarity in device efficiency between different processes indicates the broad utility of the GASP process. For completeness, we plot full device metrics in Table S3 in the Supporting Information together with representative JV and a 1‐minute stabilized power output (SPO) measurement in Figure S7a,b in the Supporting Information.

To investigate the morphology and structure of MAPbI_3_ films fabricated by GASP, we have performed X‐ray diffraction (XRD) and scanning electron microscopy (SEM) measurements. Figure 3d presents diffraction patterns recorded from a gas‐quenched spin‐control and GASP‐prepared film. The near‐identical patterns confirm that the N_2_ flow post‐treatment fully converts the precursor solution into a highly crystalline MAPbI_3_ film with no undesirable secondary phases present. SEM images indicate a similar average grain size distribution in GASP films (30 s delay) and spin‐cast films (375 nm vs 386 nm respectively). In contrast, the GASP films prepared using a delay time of 45 s between spray‐casting and application of the gas‐jet have a reduced average grain‐size of around 261 nm (see Figure S8a–d, Supporting Information). Such differences in grain size formed as a function of delay time most likely originate from the evaporation of the volatile casting solvent; here we suspect that after a 45 s delay time, the precursor solution is closer to its supersaturation point with the gas‐jet then generating a greater density of nuclei which lead to a film characterized by smaller grain sizes. Using surface profilometry, we also find that over larger areas (1 mm^2^) there is little difference in the root‐mean‐square (RMS) roughness of the spin‐cast (10.85 nm) and GASP films (10.15 nm) (see Figure S9, Supporting Information), although in both techniques we observe morphological features arising from solvent flow.

In Figure 3e, we compare the efficiency of a champion GASP‐treated MAPbI_3_ device (without any antireflective coating) with literature values for p‐i‐n and n‐i‐p architecture devices incorporating a spray‐cast active layer (data also tabulated in Table S4, Supporting Information). To our knowledge, this work represents the highest performance spray‐coated MAPbI_3_ PSC and spray‐coated p‐i‐n architecture PSC device reported to date.

### 2.4 Combining GASP Perovskite and Spray‐Coated SAMs for p‐i‐n PSCs

**Figure 4:**
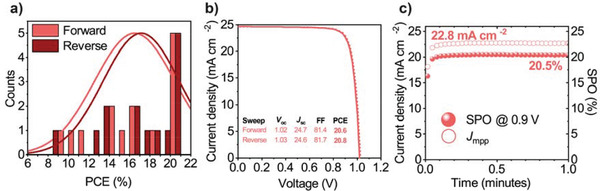
a) A histogram of device PCE for 14 cells prepared using both a spray‐coated MeO‐2PACz hole‐transporting layer and a gas‐assisted spray‐processed (GASP) MAPbI_3_ active layer, with an antireflective coating on FTO substrates. b) JV curve and c) stabilized power output (SPO) measurement for the best‐performing device.

In this section we describe the development of devices based on spray‐coated MeO‐2PACz (utilizing the optimized pre‐ and postrinsing protocol discussed above) and GASP MAPbI_3_. Again, such p‐i‐n devices utilized a thermally evaporated C_60_/BCP/Ag cathode and include an antireflective layer. In order to maximize device performance, these devices were fabricated on a fluorine‐doped tin oxide (FTO) anode as we have observed an enhancement in the *J*_sc_ for devices with an antireflective coating on FTO, rather than on ITO (see Figure S10, Supporting Information). **Figure** **4**a–c plots a histogram of device performance, together with the JV curve and SPO of the best‐performing device. Although we measure a greater spread in device performance when both the HTL and active layers are spray‐coated, high efficiency PSCs are often realized, with the champion PSC having a PCE of 20.8% (20.5% stabilized). A summary of performance metrics prepared using such “fully scalable” techniques is presented in **Table** **1**.

**Table 1:** Photovoltaic performance metrics for 14 cells comprising a spray‐coated MeO‐2PACz hole‐transporting layer and gas‐assisted spray‐processed MAPbI_3_ active layer. Here all devices are prepared on an FTO substrates and include an antireflective coating. Champion performance metrics are presented in bold, whilst the mean and standard deviation values are shown in parenthesis

Device	*J*_sc_ [mA cm^–2^]	*V*_OC_ [V]	FF [%]	PCE [%]
Spray MeO‐2PACz with GASP MAPbI_3_	**24.9** (24.1 ± 0.6)	**1.03** (0.95 ± 0.09)	**82.8** (73.0 ± 10.6)	**20.8** (16.9 ± 3.6)

We note however that our process is not under full control and there still remains a degree of variation from device to device as a result of the dip‐rinsing process (which is done by hand) which will impact on the amount of excess SAM material that is removed from the surface. We therefore attribute the distribution in device performance to partial dewetting effects of the perovskite precursor ink from the spray‐coated SAM layer, resulting in film thickness variations which likely exacerbate solvent trapping effects and leads to the formation of voids at the perovskite/glass interface.^[^ ^53^ ^]^ We anticipate that it will be possible to mitigate such effects through the use of surfactants^[^ ^80^ ^]^ or higher boiling‐point additives^[^ ^38^, ^79^ ^]^ to stabilize the interface and enable more reproducible device performance. We speculate that improvements in our process will also come by using a second spray‐head to deposit a rinse solvent and using an air‐knife to shear off the excess material.

## 3 Conclusion

We have developed two new protocols to fabricate MeO‐2PACz hole‐transporting layers; namely ultrasonic spray coating and airbrush pen coating, with both techniques realizing PSC devices having comparable performance to spin‐coated controls. Here, the use of an airbrush represents a widely accessible, high‐throughput technique to rapidly deposit carbazole‐SAM hole‐transporting materials. We then demonstrate the application of a gas‐jet quenching method to spray‐coated perovskite precursor films, creating high‐quality perovskite layers. We optimize the GASP process by control of the solid concentration in the precursor solution and the delay time between deposition of the precursor and application of the gas‐jet. Finally, we combine ultrasonic spray coating/gas‐assisted processing of both the MeO‐2PACz and MAPbI_3_ layers, creating devices having a peak PCE of 20.8% (20.5% SPO); a value that represents the highest reported stabilized efficiency for spray‐coated PSCs to date. We summarize the champion device efficiencies achieved via each processing route in Table S5 in the Supporting Information. The deposition techniques developed are ideally suited to high‐speed roll‐to‐roll fabrication of PSCs and are thus directly relevant for practical manufacture and device scale‐up.

## 4 Experimental Section

### Device Fabrication

All materials and solvents were used as received without any further purification. MeO‐2PACz (>98%), 2PACz (>98%), and PbI_2_ (99.99% trace metals basis) were purchased from Tokyo Chemical Industry. Methylammonium iodide (MAI, >99.9%), 20 mm x 15 mm ITO (≈20 Ω □^−1^) and TEC10 FTO (11–13 Ω □^−1^) were purchased from Ossila. All solvents and remaining materials including C_60_ (sublimed, 99.99%) and BCP (sublimed, 99.99%) were purchased from Sigma‐Aldrich unless otherwise stated. Substrates were patterned using a zinc and acid (4 m, HCl) etch, then cleaned by subsequent ultrasonication in diluted Helmanex solution, boiling deionized water, acetone, and isopropanol (IPA) consecutively. Substrates were then dried under a flow of N_2,_ and UV ozone treated for at least 15 min immediately prior to further processing. All perovskite solutions were filtered into a clean, glass vial through a 0.2 µm polytetrafluoroethylene (PTFE) filter prior to deposition. Note that a small increase in device performance was observed when the HTL was fabricated one day prior to processing the perovskite layer.

### Hole‐Transport Layer—Spin Coating

A stock solution of the SAM (1 mmol in ethanol) was prepared and stored under N_2_, with a small amount decanted for use as necessary. Solutions were vortex mixed for ≈30 s immediately prior to usage. 60 µL of the SAM solution was spin coated for 30 s at 3000 rpm in a N_2_ filled glovebox, followed by annealing at 100 °C for 10 min. No subsequent rinsing steps were applied.

### Hole‐Transport Layer—Dip Coating

SAM solutions (0.1 mmol in ethanol) were prepared by diluting a quantity of the stock solution. Dip coating was conducted in a polypropylene beaker to minimize usage of material. Cleaned substrates were immersed in the solution overnight under ambient conditions, with the beaker sealed using Parafilm. After deposition, excess solution was removed with a N_2_ gun. Substrates were then moved into the glovebox and annealed at 100 °C for 10 min. After annealing, dip‐coated substrates were rinsed three times by successive submersion in ethanol and then dried using a N_2_ flow.

### Hole‐Transport Layer—Spray Coating

SAM solutions (0.1 mmol in ethanol) were prepared by diluting the stock solution. Spray coating was performed using a Sonotek Exactacoat system mounted with an Impact spray head. The SAM solution was delivered through a vibrating piezoelectric tip at a flow rate of 1.5 mL min^–1^. The spray head was moved in three passes over the substrate surface at a separation of around 3 cm at a speed of 40 mm s^–1^. Optimized performance was achieved by subjecting spray‐coated substrates to a single rinse performed immediately after the deposition of the SAM by submersion in ethanol whilst the film was still wet. Substrates were then dried under a N_2_ flow and annealed at 100 °C for 10 min. After annealing, the substrates were rinsed three times by successive submersion in ethanol and then dried with a N_2_ flow.

### Hole‐Transport Layer—Airbrush Coating

SAM solutions (0.1 mmol in ethanol) were prepared by diluting the stock solution. Solutions were loaded into an airbrush pen located in a N_2_ filled glovebox. Substrates were spray coated until a uniform, wet film was created. They were then annealed at 100 °C for 10 min. After annealing, the airbrush‐coated substrates were rinsed three times by successive submersion in ethanol and then dried with a N_2_ flow.

### Perovskite—Spin Coating

An MAPbI_3_ (1 m) precursor in 2‐ME was prepared and deposited according to a previously reported protocol.^[^ ^20^ ^]^ Briefly, PbI_2_ (461 mg mL^–1^) and MAI (159 mg mL^–1^) were dissolved in 2‐ME by combination of vortex mixing and stirring. DMSO was introduced as an additive at a concentration of ≈11.77 mol%. 60 µL of the precursor solution was dropped onto the center of the substrate before it was spun at 800 rpm s^–1^ for 5 s, followed by 4000 rpm for 35 s. A N_2_ flow of around 20 psi was directed at the substrate starting 6 s after the beginning of the spin program. The MAPbI_3_ films were then annealed at 100 °C for 10 min.

### Perovskite—Spray Coating

2‐ME MAPbI_3_ (0.5–1 m) precursor solutions were deposited using a Sonotek Exactacoat System. The solution was delivered at a flow rate of 1.5 mL min^–1^ to the Impact head which operated at 2 W. During coating, the spray head moved over the substrate at a distance of around 3 cm at a speed of 80 mm s^–1^. No heating was applied to the substrate during deposition. After deposition of the perovskite precursor, an automated gantry passed an air knife (Meech A8 80 mm Air Knife, RS Components) over the substrate at a speed of 3 mm s^–1^ and a distance of around 2 cm from the surface. This delivered an N_2_ flow at 20 psi to the surface at an angle of 45° from the substrate normal. This process occurred after a delay time of between 5 and 60 s after spray coating, with a delay of 30 s corresponding to the optimized process. Perovskite films were then annealed at 100 °C for 10 min.

### Electron‐Transport Layer and Cathode

The substrates were transferred to a thermal evaporator (Angstrom Engineering). BCP (8 nm), C_60_ (23 nm), and silver (100 nm) were deposited sequentially through shadow masks without breaking vacuum. During evaporation, the chamber base pressure was maintained at < 2.4 × 10^–6^ mbar. BCP and C_60_ were deposited using alumina crucible sources (RADAK, Luxel Corp.) at a constant rate of 0.1 Å s^–1^_._ It was found that the C_60_ became discolored after each evaporation run and was therefore replaced with fresh material each time. Silver pellets (Lesker) were deposited from resistive sources at a rate of 0.1–1.0 Å s^–1^. Following evaporation, devices were encapsulated using an epoxy pen (Bluefixx Blue LED Repair Pen, Combined Precision Components) and glass encapsulation coverslips (Ossila). For some devices, an antireflective coating of LiF (Lithium Fluoride, 100 nm) was applied to the substrate back‐surface by thermally evaporating at 1 Å s^–1^ at a base pressure of at least 4 × 10^–6^ mbar.

### Device Characterisation

JV measurements were recorded under ambient conditions using a Newport 92251A‐1000 solar simulator. No preconditioning of devices was carried out. Prior to testing, the Air Mass 1.5 (AM1.5) spectrum was adjusted to 100 mW cm^–2^ at the substrate holder location using an National Renewable Energy Laboratory (NREL) certified silicon reference cell. The active measurement area was defined using metal aperture masks with a calibrated area of 2.5 mm^2^. A Keithley 237 source‐measure unit swept devices between −0.1 and 1.2 V at 100 mV s^–1^. SPO measurements were performed by holding the device at a bias defined by the average voltage at maximum power (*V*_mpp_) determined from the forward and reverse sweeps. Note that an increase in device PCE was frequently observed over the first few days after fabrication.

A Bruker DekTak XT surface profilometer was used to investigate large‐area morphology of the thin films deposited. A stylus (12.5 µm radius tip) was raster scanned over the surface (1000 µm lines each separated by 1 µm) using a stylus force of 3 mg. The resultant line scans were stitched together to form a large‐area topographical “map”. Map profiles were processed using Gwyddion software to remove artificial curvature and step line correct in both the *x* and *y* axis. Roughness measurements were extracted using the same software.

External quantum efficiency measurements were recorded over a 325–900 nm range using a Newport QuantX‐300 Quantum Efficiency Measurement System. The system was equipped with a 100 W Xenon arc lamp focused through an Oriel Monochromator (CS130B) and chopped at 25 Hz.

XRD data were recorded at room temperature using a PANalytical X'Pert Pro system equipped with a Copper Line Focus X‐ray tube run at 45 kV with a tube current of 40 mA. The diffractometer operated in Bragg–Brentano geometry to record diffraction patterns from 5° to 100° 2*θ*.

SEM top‐down images of the perovskite surface were recorded using a Field Emission Gun (FEG) Raith SEM at a working distance of ≈4.5 mm and beam energy of 1.5 kV. Grain size analysis was performed using ImageJ software.

Atomic Force Microscopy (Veeco Dimension 3100) samples were imaged in Intermittent Contact (Tapping) Mode with a NuNano Scout 350 cantilever (nominal spring constant 42 N m^−1^, resonant frequency 350 kHz). Each sample was scanned over two 10 × 10 µm^2^ areas with a resolution of 512 × 512 pixels.

## Conflict of Interest

D.G.L. is a director of the materials science company Ossila that retails materials and equipment for thin film fabrication, including for research and development of perovskite photovoltaics.

## Supporting information

Supporting Information

## Data Availability

The data that support the findings of this study are available from the corresponding author upon reasonable request.
