# Supplementary material for: Gas‐Assisted Spray Coating of Perovskite Solar Cells Incorporating Sprayed Self‐Assembled Monolayers
Source: Adv Sci (Weinh). 2022 Feb 9;9(14):2104848. doi: 10.1002/advs.202104848 (PMC9108661; doi:10.1002/advs.202104848)
Supplement: Supplementary file 1 — Supporting Information [file ADVS-9-2104848-s001.pdf]

ADVANCED SCIENCE
Open Access

Supporting Information

for *Adv. Sci.*, DOI 10.1002/advs.202104848

Gas-Assisted Spray Coating of Perovskite Solar Cells Incorporating Sprayed Self-Assembled Monolayers

*Elena J. Cassella, Emma L. K. Spooner, Timothy Thornber, Mary E. O'Kane, Thomas E. Catley, James E. Bishop, Joel A. Smith, Onkar S. Game and David G. Lidzey**

ADVANCED SCIENCE
Open Access

Supporting Information

for *Adv. Sci.*, DOI: 10.1002/advs.202104848

Gas-Assisted Spray Coating of Perovskite Solar Cells Incorporating Sprayed Self-Assembled Monolayers

*Elena J. Cassella, Emma L.K. Spooner, Timothy Thornber, Mary E. O'Kane, Thomas E. Catley, James E. Bishop, Joel A. Smith, Onkar S. Game, David G. Lidzey**

Supporting Information

Gas-Assisted Spray Coating of Perovskite Solar Cells Incorporating Sprayed Self-Assembled Monolayers

*Elena J. Cassella, Emma L.K. Spooner, Timothy Thornber, Mary E. O'Kane, Thomas E. Catley, James E. Bishop, Joel A. Smith, Onkar S. Game, David G. Lidzey**

Table of contents

1. Optimisation of spray coated MeO-2PACz

1.1. Effect of solution flow rate and number of spray passes

1.2. Contact angle of DMF:DMSO and water on MeO-2PACz

1.3. Investigating a pre-anneal rinsing step

1.4. Effect of rinsing on surface roughness

1.5. Summary of optimized dip rinsing protocols for each SAM deposition method

1.6. Tabulated device data for Figure 2

2. Development of GASP procedure

2.1. Schematic illustration of the perovskite spray processing steps

2.2. Effect of precusor concentration on perovskite: glass interface

2.3. Solar cell performance metrics for Figure 3 data

2.4. Additional thin film characterisation of GASP-fabricated PSCs

2.4.1. SEM images

2.4.2. DekTak mapping scans

2.5. State-of-the-art spray-coated $MAPbI_3$ perovskite solar cells

3. Additional data

3.1. Improved device performance arising from FTO substrates

3.2. Champion devices summary table

1. Optimisation of spray-coated MeO-2PACz

1.1. Effect of SAM solution flow rate and number of spray passes

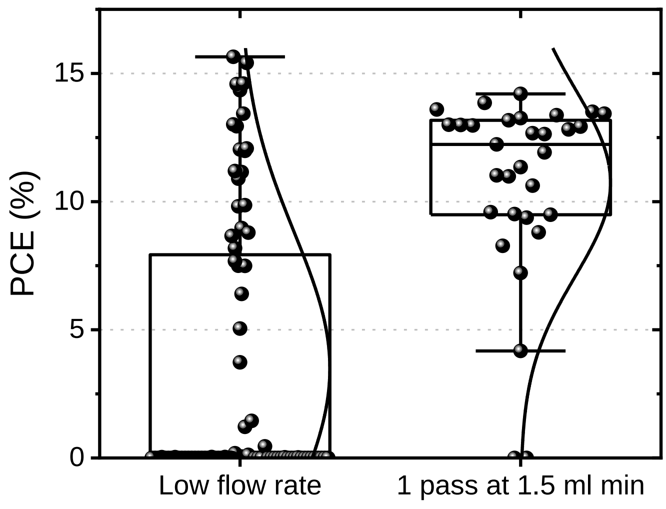

Figure S1: Device performance metrics for sprayed MeO-2PACz films at low flow rate (1 ml min^{-1}), at which a wet film is not achieved and at 1.5 ml min^{-1} with only a single pass of the spray head, where a wet film is realized. Both cases yield lower, more widely distributed device performance than spin coated MeO-2PACz devices.

1.2 Contact angle of DMF:DMSO and deionised water on MeO-2PACz

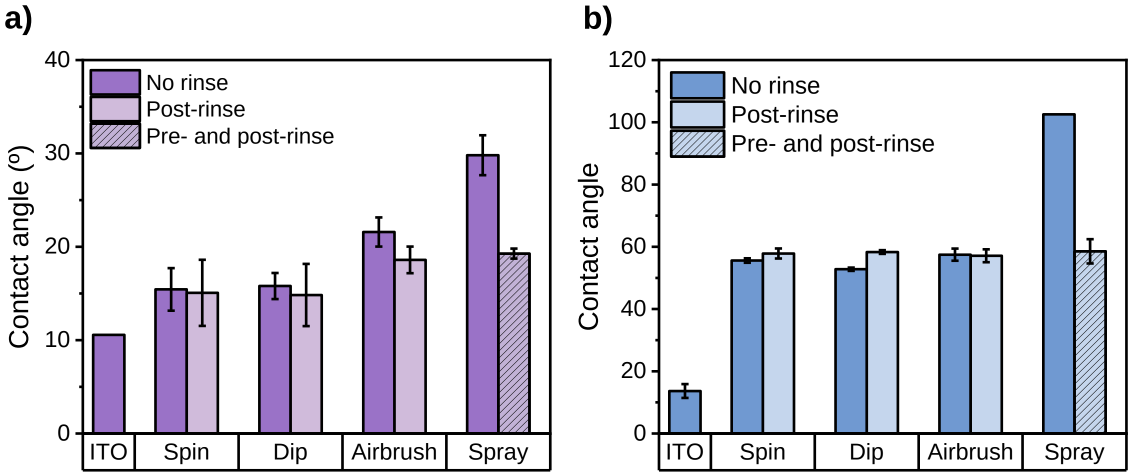

Figure S2: The initial contact angles of (a) a 4:1 DMF: DMSO solution and (b) deionised water on non-rinsed (darker) and post-anneal rinsed (lighter) or pre- and post-rinsed (hatched) films of MeO-2PACz fabricated by either spin, dip, airbrush, or spray coating. The optimized dip-rinsing protocols used here successfully reduce the contact angle of both solutions on spray and airbrushed MeO-2PACz to a similar level as those for spin coated control films.

1.3 Investigating a pre-annealing rinsing step

The following discussion refers to the effect of a pre-rinse process on device efficiency and the dewetting of the perovskite film. Note however, that the optimized process discussed in the main paper includes both a pre- and post-anneal rinse.

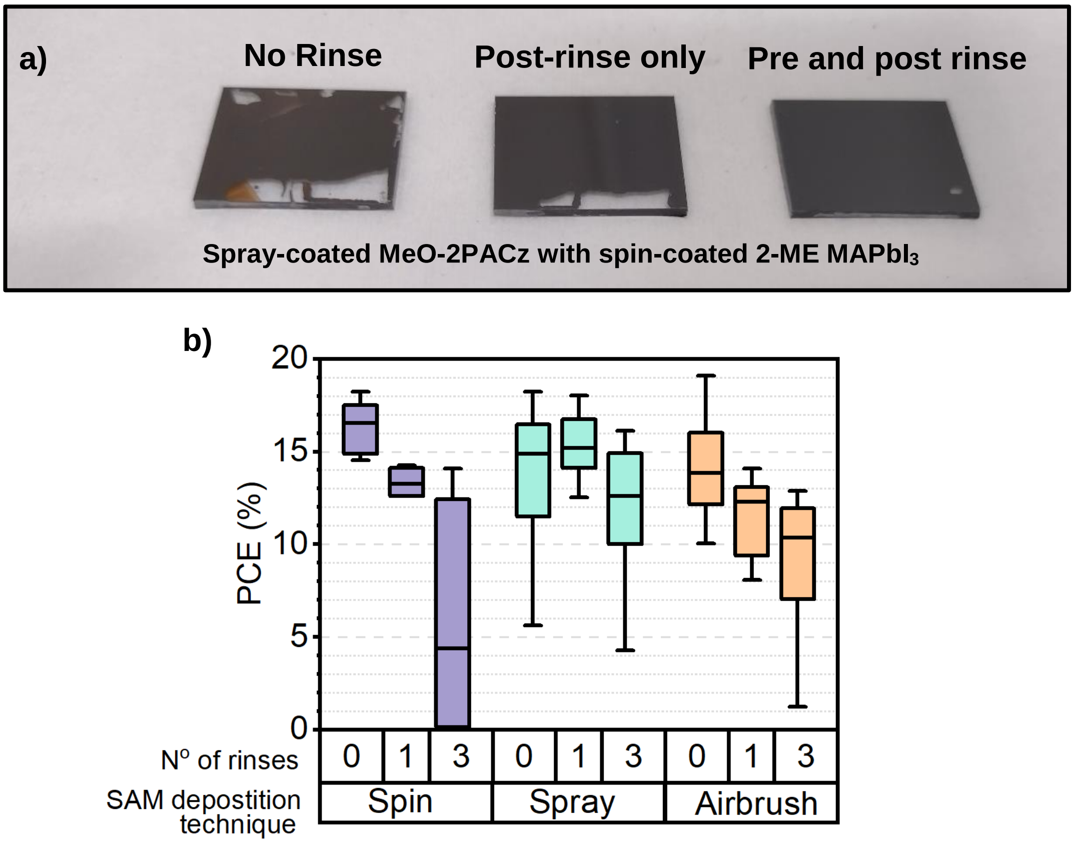

Figure S3: (a) Photographs of $MAPbI_3$ films spun from 2-methoxy ethanol on spray-coated MeO-2PACz with (left) no rinsing, (middle) post-anneal rinsing only, and (right) with both pre- and post-anneal rinsing. It can be seen the wetting behaviour of the spin-coated 2-methoxyethanol ink mostly overcomes the dewetting effects arising from the excess sprayed material.

(b) The PCE of spin- (purple) spray- (green) and airbrush- (orange) coated MeO-2PACz with 0, 1 and 3 repeated pre-rinse steps. In all cases, the $MAPBI_3$ perovskite was spin-coated. For all conditions, the application of 3 pre-rinses were detrimental to device performance.

As can be seen in Figure S3(b), when no rinses are applied to the spray-coated SAM, we see a slight drop in device performance compared to devices that utilise a spin-coated SAM that have also not been rinsed. Devices made on unrinsed, spray-cast SAMs also have a larger statistical spread in their efficiency. Devices fabricated using spin-cast perovskite films on the un-rinsed spray-cast SAM layer still have high performance despite a noticeable degree of dewetting observed in the perovskite films (see left-hand image in Figure S3(a)).

When only the post-annealing rinse protocol is applied (middle image, Figure S3(a)), some slight dewetting is seen. Applying a single pre-rinsing step to the spray-cast SAM alone significantly reduces this spread in device performance (see Figure S3(b)), but the median device efficiency is lower than spin-coated, unrinsed control devices due to some residual dewetting effects. We note that for techniques such as spray coating, where there are no additional forces to encourage coalescence of the wet as-deposited film, these dewetting effects on spray-coated MeO-2PACz without any rinsing, or with only post-annealing rinsing, prevent the 2-methoxy ethanol precursor from forming a continuous film and devices *cannot* be fabricated. This therefore necessitates additional rinsing steps, further to either single pre-rinsing, or post-rinsing alone.

Any pre-anneal rinsing applied to spin- or airbrush- coated SAMs (see Figure S3(b)) reduces device performance, likely from over-removing material and resulting in contact between the perovskite and ITO surface.

1.4. Effect of rinsing on surface roughness

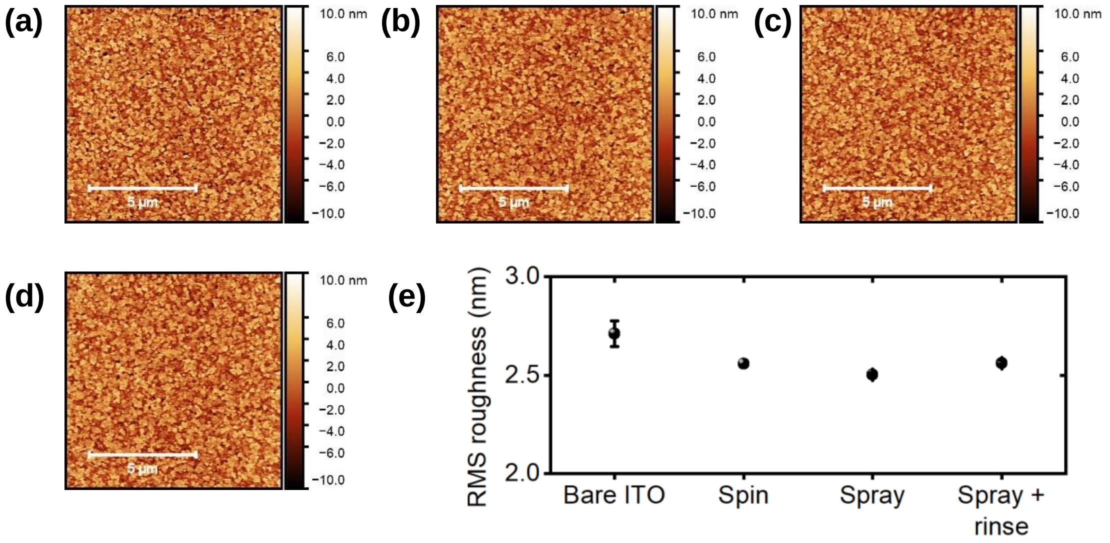

Figure S4: 10 µm x 10 µm AFM-scans of (a) bare ITO, (b) ITO with spin-coated MeO-2PACz and (c) ITO with spray-coated MeO-2PACz, and (d) ITO with spray-coated MeO-2PACz both pre- and post-rinsed. The plot in (e) shows the average (n=2) root-mean-square roughness (R_q) of each sample. It can be seen that there is very little visual difference between samples. We observe a small trend in roughness reduction in the MeO-2PACz coated samples with the R_q of the bare ITO being slightly greater that of ITO coated with MeO-2PACz (2.71 and 2.50 nm respectively). However, we do not believe such an observation has statistical significance. We conclude that the thin MeO-2PACz layers conformally coat the ITO surface, with the AFM being unable to resolve significant changes in roughness from the polycrystalline ITO below.

1.5. Summary of the optimized rinsing protocol for each deposition method

Table S1: A summary of the optimized rinsing protocol developed for each MeO-2PACz film fabrication technique.

MeO-2PACz deposition technique	Pre-anneal rinse	Post-anneal rinse
Spin	None	None
Dip	None	3x
Airbrush	None	3x
Spray	1x	3x

1.6. Tabulated device data for Figure 2

Table S2a: A summary of PSC performance metrics (including both forward and reverse JV sweeps, and data for devices **without anti-reflective coatings on ITO**) for spin coated $MAPbI_3$ perovskite on MeO-2PACz deposited via each deposition technique. Bold font data represents champion performance metrics, while averages and standard deviations are shown in parentheses.

SAM deposition technique	J_{sc} [mA cm^{-2}]	V_{OC} [V]	FF [%]	PCE [%]	N_{cells}
**Spin**	**23.8** (22.7 ± 0.8)	**1.06** (1.01 ± 0.05)	**76.3** (70.0 ± 6.2)	**18.2** (16.2 ± 2.2)	**18**
**Dip**	**23.9** (21.7 ± 1.2)	**1.04** (1.01 ± 0.04)	**77.5** (72.6 ± 7.0)	**18.6** (15.9 ± 2.1)	**25**
**Spray**	**23.5** (23.0 ± 0.4)	**1.04** (1.01 ± 0.02)	**77.1** (75.6 ± 1.6)	**18.4** (17.6 ± 0.6)	**6**
**Airbrush**	**23.0** (22.9 ± 0.3)	**1.03** (1.02 ± 0.01)	**76.7** (73.6 ± 2.0)	**18.1** (17.3 ± 0.6)	**7**

Table S2b: A summary of PSC performance metrics (including both forward and reverse JV sweeps, and data for devices **with anti-reflective coatings on ITO**) for spin coated $MAPbI_3$ perovskite on MeO-2PACz deposited via each deposition technique. Bold font data represents champion performance metrics, while averages and standard deviations shown in parentheses.

SAM deposition technique	J_{SC} [mA cm^{-2}]	V_{OC} [V]	FF [%]	PCE [%]	N_{cells}
**Spin**	**24.6** (24.0 ± 0.5)	**1.05** (1.03 ± 0.02)	**83.8** (81.5 ± 1.9)	**20.7** (20.1 ± 0.9)	**9**
**Spray**	**23.6** (23.0 ± 0.4)	**1.04** (1.00 ± 0.05)	**84.3** (81.8 ± 2.7)	**20.3** (18.6 ± 1.3)	**22**
**Airbrush**	**23.5** (22.9 ± 0.6)	**1.03** (1.00 ± 0.02)	**83.0** (80.6 ± 2.4)	**19.9** (18.4 ± 1.1)	**18**

2. Development of GASP procedure

2.1. Schematic illustration of the perovskite spray coating process

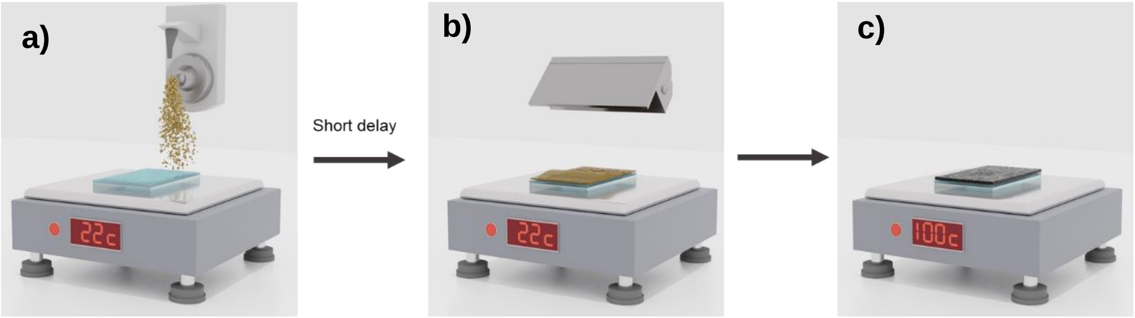

Figure S5: (a) The gantry moves the spray head linearly over the (unheated) substrate surface. The focusing gas directs the wet droplets to the substrate surface. (b) After a short delay – to enable coalescence of the deposited droplets – the gantry moves the air knife over the surface, blowing room temperature nitrogen at 45° to the normal over the wet film. (c) The films are then annealed at 100 °C for 10 minutes.

2.2. Effect of precusor concentration on perovskite: glass interface

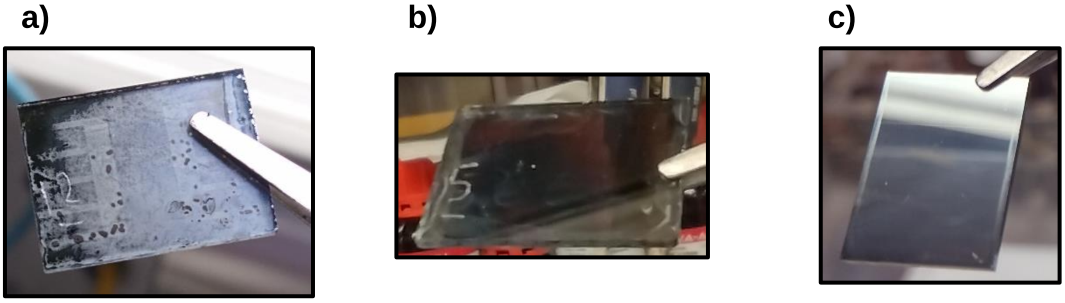

Figure S6: Photographs of (a) the rear side of an ITO substrate coated with spin-coated MeO-2PACz and GASP $MAPbI_3$ at 1 M concentration, wherein the cloudy appearance is indicative of voids between the perovskite and the glass surface. (b) The rear side of an ITO substrate coated with spin-coated MeO-2PACz and GASP $MAPbI_3$ at 0.5 M concentration without any noticeable voids, and (c) the top surface of the same substrate, showing a highly specular surface.

2.3. Solar cell performance metrics for Figure 3 data

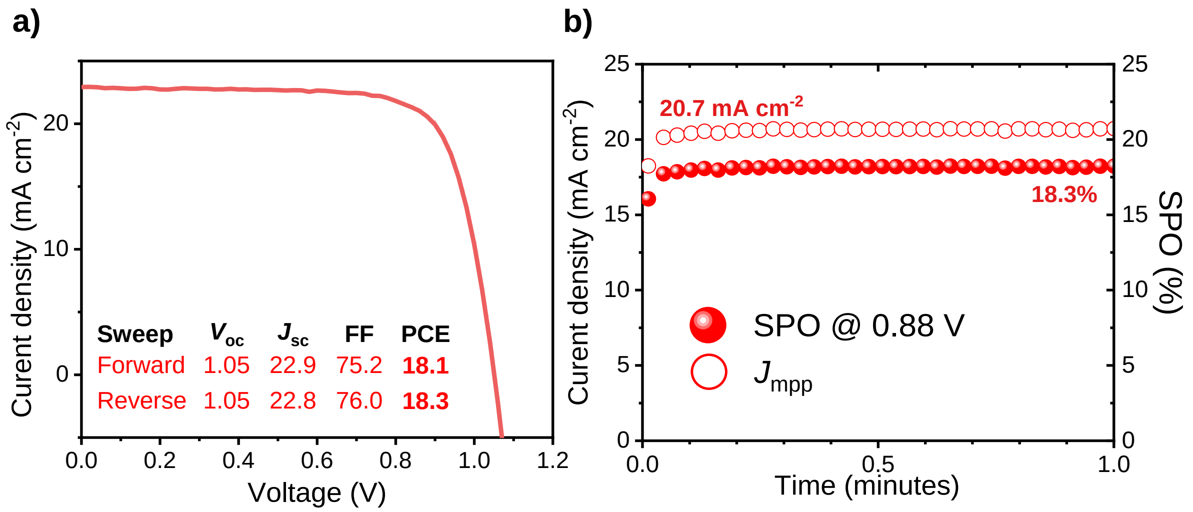

Figure S7: (a) JV curve and (b) stabilized power output (SPO) for the best-performing gas-assisted spray-processed (GASP) $MAPbI_3$ on spin-coated MeO-2PACz without an anti-reflective coating on ITO substrates.

Table S3: Device statistics for devices (**without anti-reflective coatings on ITO**) fabricated with spin-coated MeO-2PACz and $MAPbI_3$ (19 cells) vs. spin-coated MeO-2PACz and GASP $MAPbI_3$ (23 cells). Champion performance data is in boldface type, with mean and standard deviations parenthesized.

$MAPbI_3$ deposition technique	J_{sc} [mA cm^{-2}]	V_{OC} [V]	FF [%]	PCE [%]
**Spin**	**23.8**<br>(22.7 ± 0.9)	**1.06**<br>(1.02 ± 0.06)	**76.3**<br>(70.1 ± 6.0)	**18.2**<br>(16.2 ± 2.1)
**GASP**	**23.0**<br>(22.1 ± 0.4)	**1.07**<br>(1.05 ± 0.03)	**76.1**<br>(72.4 ± 2.8)	**18.3**<br>(17.0 ± 1.0)

2.4. Additional thin film characterisation of GASP-treated PSCs

2.4.1. SEM images

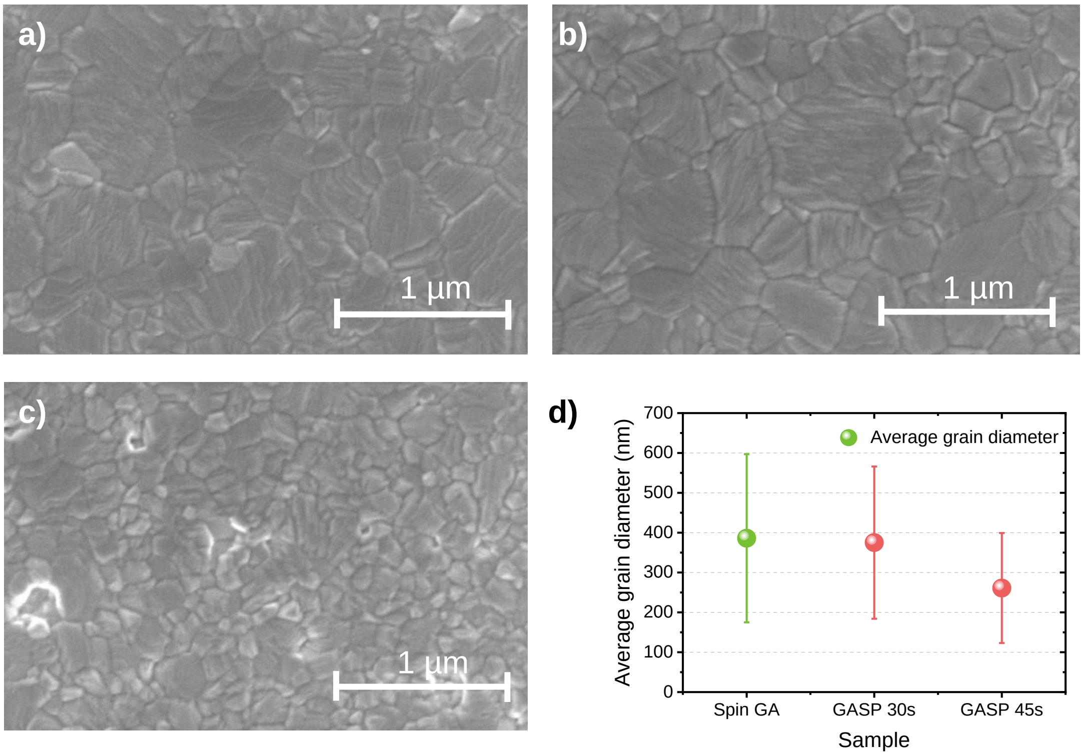

Figure S8: Scanning electron microscope (SEM) images of (a) gas-assisted spin-coated $MAPbI_3$, (b) GASP $MAPbI_3$ with 30 s delay between deposition and airflow, and (c) GASP $MAPbI_3$ with 45 s delay between deposition and airflow. 1 µm scale bar inset. (d) The mean and standard deviation of grain sizes for each sample, extracted with Image J software. The grain structure for 30 s delay GASP films is similar to spin controls, however at a delay time of 45 s, there is a greater number of smaller grains.

2.4.2. Dektak profilometer topographical mapping scans

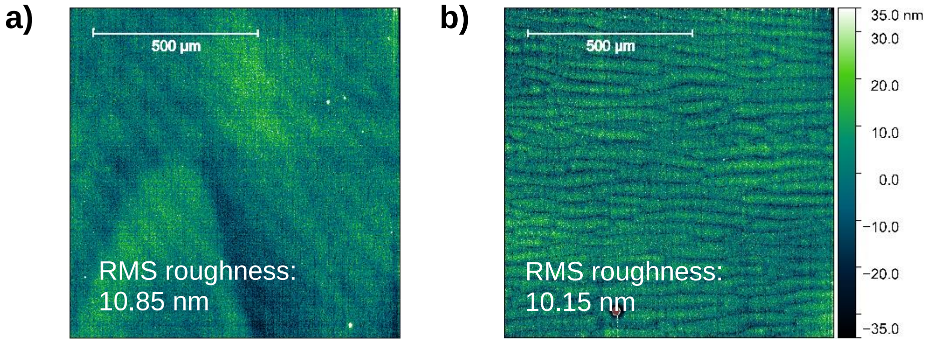

Figure S9: 1 mm x 1 mm profilometer mapping scans of (a) gas-assisted spin-coated $MAPbI_3$ and (b) GASP (30 s delay) $MAPbI_3$ films with 500 µm scale bar inset. There is little disparity in the root-mean-square (RMS) roughness between the films.

2.5. State-of-the-art spray-coated $MAPbI_3$ perovskite solar cells

Table S4: State-of-the-art spray coated inverted and n-i-p $MAPbI_3$ PSCs.

Year	PCE [%]	J_{sc} [mA cm^{-2}]	V_{oc} [V]	FF [%]	Config.	Stack	Ref
2014	11.1	16.8	0.92	72	p-i-n	ITO/PEDOT:PSS/$MAPbI_{3-x}Cl_x$/PCBM/Ca/Al	[1]
2015	10.2	17.3	0.93	0.63	p-i-n	ITO/PEDOT:PSS/$MAPbI_3$/C_{60}/BCP/Al	[2]
2015	6.93	23.01	0.69	43.4	n-i-p	FTO/TiO_2/$MAPbI_3$/Au	[3]
2015	12.5	19.1	0.948	69	n-i-p	FTO/c-TiO_2/mp-TiO_2/$MAPbI_3$/spiro-OMeTAD/Ag	[4]
2016	15.7	22.5	0.95	73	n-i-p	ITO/TiO_2/$MAPbI_{3-x}Br_x$/Spiro-OMeTAD/Au	[5]
2016	16.03	20.27	1.047	75.5	n-i-p	FTO/c-TiO_2/mp-TiO_2/$MAPbI_3$/spiro-OMeTAD/Au	[6]
2016	11.4	17.6	0.92	74	p-i-n	ITO/PEDOT:PSS/$MAPbI_{3-x}Cl_x$/PCBM/LiF/Al	[7]
2017	13	19	0.99	69	n-i-p	FTO/c-TiO_2/np-TiO_2/$MAPbI_3$/spiro-MeOTAD/Au	[8]
2017	13.27	17.79	0.98	74.7	p-i-n	ITO/PEDOT:PSS/$MAPbI_3$/C_{60}/BCP/LiF/Al	[9]
2017	12.01	22.4	1.03	52	n-i-p	FTO/c-TiO_2/mp-TiO_2/$MAPbI_3$/spiro-OMeTAD/Au	[10]
2017	13.35	20.8	0.87	73.6	p-i-n	FTO/PEDOT:PSS/$MAPbI_{3-x}Br_x$/PCBM/BCP/Ag	[11]
2017	15.4	21	0.96	76	p-i-n	ITO/PEDOT:PSS/$MAPbI_3$/C_{60}/PCBM/Al	[12]
2017	12.6	22.5	0.87	63.4	p-i-n	ITO/PEDOT:PSS/$MAPbI_3$/PCBM/Ag	[13]
2018	14.2	22.4	0.92	69	p-i-n	ITO/PEDOT:PSS/$MAPbI_{3-x}Cl_x$/PCBM/PEIE/Ag	[14]
2018	17.3	21.70	1.033	73	n-i-p	FTO/SnO_2/$MAPbI_3$/Spiro-OMeTAD/MoO_x/Al	[15]
2018	12.3	21.94	0.83	67.6	p-i-n	ITO/PEDOT:PSS/$MAPbI_3$/C_{60}/Ag	[16]
2018	16.9	21.6	1.03	76	p-i-n	ITO/PEDOT:PSS/$MAPbI_3$/C_{60}/BCP/Cu	[17]
2019	15.61	22.27	1.00	70.3	p-i-n	FTO/NiOx/$FA_{0.25}MA_{0.75}PbI_3$/C_{60}/BCP/Ag	[18]
2020	13.76	22.31	0.98	62.9	p-i-n	ITO/NiO_x/$MAPbI_3$/C_{60}/PCBM:PNDI(2OD)T2/Ag	[19]
2020	18	-	-	-	p-i-n	ITO/NiO_x/$(Cs_{0.17}FA_{0.83})PbI_3$/$C_{60}$/BCP/Ag	[20]
2020	17.11	21.81	1.03	76	p-i-n	ITO/NiO_x/$MAPbI_{3-x}Br_x$/C_{60}/BCP/Ag	[21]
2021	6.18$^{a)}$	2.91	37.2	59.4	p-i-n	ITO/NiO_x/$MAPbI_3$/C_{60}/BCP/Ag	[22]
**2021**	**18.3**	**22.8**	**1.05**	**76**	**p-i-n**	**ITO/MeO-2PACz/$MAPbI_3$/C_{60}/BCP/Ag**	**This work**

$^{a)}$ 10.4 cm^2 minimodule

3. Additional data

3.1. Improved device performance arising from FTO substrates

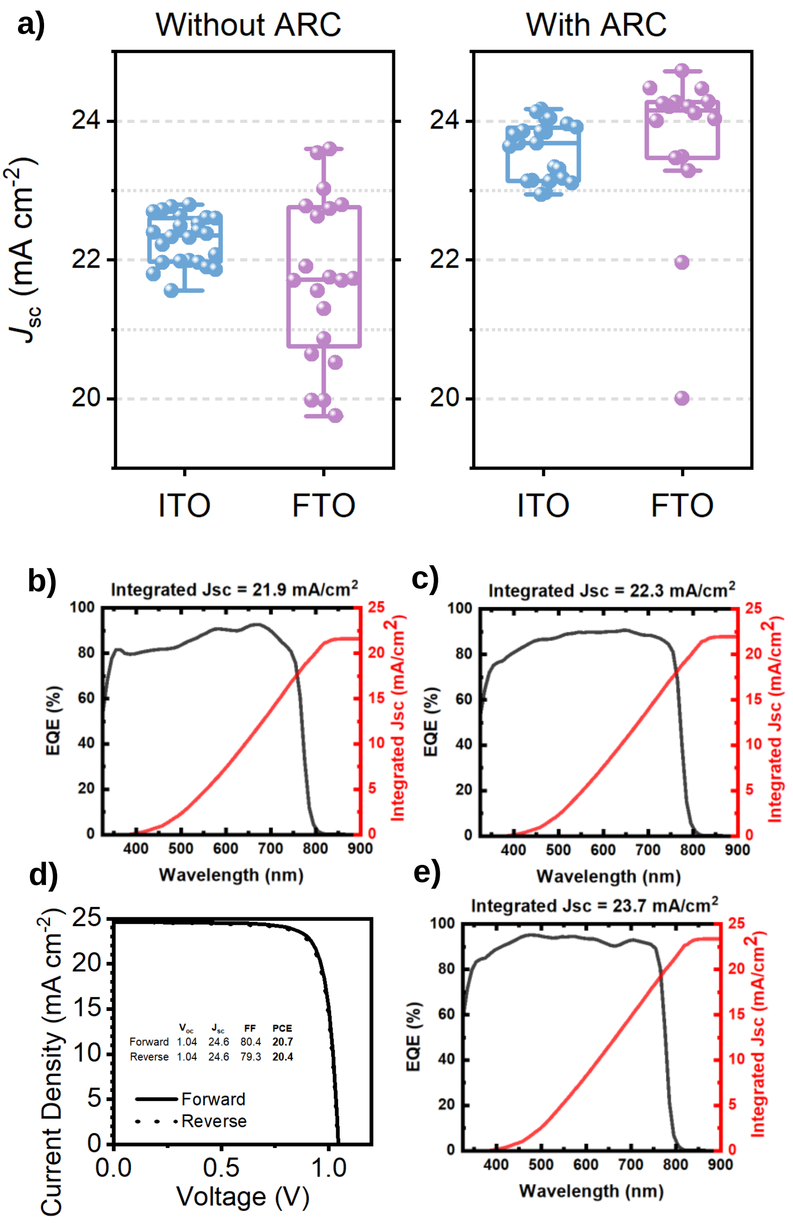

	V_{oc}	J_{sc}	FF	PCE
Forward	1.04	24.6	80.4	20.7
Reverse	1.04	24.6	79.3	20.4

Figure S10: (a) The J_{sc} of $MAPbI_3$ devices on both ITO and FTO without (left) and with (right) an anti-reflective coating applied to the substrate surface. The FTO-based devices have a higher J_{sc} than ITO-based devices after application of the coating. We find that the EQE of the ITO substrates (b) has a lower contribution to charge generation in the 400 to 600 nm region than the FTO substrates (c). (d) and (e) present the J-V curve and EQE spectra of a best-performing spin-coated control device on FTO substrate with the anti-reflective coating applied.

3.2. Champion devices summary table

Table S5: A summary of best-performing devices for each case presented within this work alongside corresponding control device performance.

**MeO-2PACz deposition technique**	Spin	Spin	Spray (with pre- and post-rinse)	Spin	Spray (with pre- and post- rinse)
**$MAPbI_3$ deposition technique**	Spin	Spin	Spin	GASP	GASP
**Manuscript Section**	2.2 and 2.3	2.4	2.2	2.3	2.4
**Substrate**	ITO	FTO	ITO	ITO	FTO
**Anti-reflective coating**	X (✓)	✓	X (✓)	X	✓
**PCE (%)**	**18.2 (20.7)**	**20.7**	**18.4 (20.2)**	**18.3**	**20.8**

References

[1] A. T. Barrows, A. J. Pearson, C. K. Kwak, A. D. F. Dunbar, A. R. Buckley, D. G. Lidzey, *Energy Environ. Sci.* **2014**, *7*, 2944.

[2] M. Ramesh, K. M. Boopathi, T. Y. Huang, Y. C. Huang, C. S. Tsao, C. W. Chu, *ACS Appl. Mater. Interfaces* **2015**, *7*, 2359.

[3] S. Gamliel, A. Dymshits, S. Aharon, E. Terkieltaub, L. Etgar, *J. Phys. Chem. C* **2015**, *119*, 19722.

[4] F. Li, C. Bao, H. Gao, W. Zhu, T. Yu, J. Yang, G. Fu, X. Zhou, Z. Zou, *Mater. Lett.* **2015**, *157*, 38.

[5] J. G. Tait, S. Manghooli, W. Qiu, L. Rakocevic, L. Kootstra, M. Jaysankar, C. A. Masse De La Huerta, U. W. Paetzold, R. Gehlhaar, D. Cheyns, P. Heremans, J. Poortmans, *J. Mater. Chem. A* **2016**, *4*, 3792.

[6] H. Huang, J. Shi, L. Zhu, D. Li, Y. Luo, Q. Meng, *Nano Energy* **2016**, *27*, 352.

[7] D. K. Mohamad, J. Griffin, C. Bracher, A. T. Barrows, D. G. Lidzey, *Adv. Energy Mater.* **2016**, *6*, 1.

[8] M. Remeika, S. R. Raga, S. Zhang, Y. Qi, *J. Mater. Chem. A* **2017**, *5*, 5709.

[9] S. C. Hong, G. Lee, K. Ha, J. Yoon, N. Ahn, W. Cho, M. Park, M. Choi, *ACS Appl. Mater. Interfaces* **2017**, *9*, 7879.

[10] S. Kavadiya, D. M. Niedzwiedzki, S. Huang, P. Biswas, *Adv. Energy Mater.* **2017**, *7*, 1.

[11] G. Chai, S. Luo, H. Zhou, W. A. Daoud, *Mater. Des.* **2017**, *125*, 222.

[12] S. Bag, J. R. Deneault, M. F. Durstock, *Adv. Energy Mater.* **2017**, *7*, 1.

[13] J. Yao, L. Yang, F. Cai, Y. Yan, R. S. Gurney, D. Liu, T. Wang, *Sustain. Energy Fuels* **2018**, *2*, 436.

[14] S. Han, H. Kim, S. Lee, C. Kim, *ACS Appl. Mater. Interfaces*, **2018**, *10*, 7281.

[15] S. Ulična, B. Dou, D. H. Kim, K. Zhu, J. M. Walls, J. W. Bowers, M. F. A. M. Van Hest, *ACS Appl. Energy Mater.* **2018**, *1*, 1853.

[16] L. H. Chou, X. F. Wang, I. Osaka, C. G. Wu, C. L. Liu, *ACS Appl. Mater. Interfaces* **2018**, *10*, 38042.

[17] M. Park, W. Cho, G. Lee, S. C. Hong, M. cheol Kim, J. Yoon, N. Ahn, M. Choi, *Small* **2019**, *15*, 1.

[18] Y. S. Chou, L. H. Chou, A. Z. Guo, X. F. Wang, I. Osaka, C. G. Wu, C. L. Liu, *ACS Sustain. Chem. Eng.* **2019**, *7*, 14217.

[19] L. H. Chou, Y. T. Yu, X. F. Wang, I. Osaka, C. G. Wu, C. L. Liu, *Energy Technol.* **2020**, *8*, 1.

[20] N. Rolston, W. J. Scheideler, A. C. Flick, J. P. Chen, H. Elmaraghi, A. Sleugh, O. Zhao, M. Woodhouse, R. H. Dauskardt, *Joule* **2020**, *4*, 2675.

[21] A. Z. Guo, L. H. Chou, S. H. Yang, D. Wang, X. F. Wang, I. Osaka, H. W. Lin, C. L. Liu, *Adv. Mater. Interfaces* **2021**, *8*, 1.

[22] L. H. Chou, Y. T. Yu, I. Osaka, X. F. Wang, C. L. Liu, *J. Power Sources* **2021**, *491*, 229586.
